# Role of cardiopulmonary interactions in development of ventilator-induced lung injury—Experimental evidence and clinical Implications

**DOI:** 10.3389/fphys.2023.1228476

**Published:** 2023-07-18

**Authors:** Neel Shah, Bhushan H. Katira

**Affiliations:** Division of Pediatric Critical Care Medicine, Department of Pediatrics, Washington University in St Louis, St Louis, MO, United States

**Keywords:** vascular shearing, endothelial injury, VILI (ventilator induced lung injury), lung deflation injury, cardiopulmonary interactions

## Abstract

Ventilator-induced lung injury (VILI) impacts outcomes in ARDS and optimization of ventilatory strategies improves survival. Decades of research has identified various mechanisms of VILI, largely focusing on airspace forces of plateau pressure, tidal volume and driving pressure. Experimental evidence indicates the role of adverse cardiopulmonary interaction during mechanical ventilation, contributing to VILI genesis mostly by modulating pulmonary vascular dynamics. Under passive mechanical ventilation, high transpulmonary pressure increases afterload on right heart while high pleural pressure reduces the RV preload. Together, they can result in swings of pulmonary vascular flow and pressure. Altered vascular flow and pressure result in increased vascular shearing and wall tension, in turn causing direct microvascular injury accompanied with permeability to water, proteins and cells. Moreover, abrupt decreases in airway pressure, may result in sudden overperfusion of the lung and result in similar microvascular injury, especially when the endothelium is stretched or primed at high positive end-expiratory pressure. Microvascular injury is universal in VILI models and presumed in the diagnosis of ARDS; preventing such microvascular injury can reduce VILI and impact outcomes in ARDS. Consequently, developing cardiovascular targets to reduce macro and microvascular stressors in the pulmonary circulation can potentially reduce VILI. This paper reviews the role of cardiopulmonary interaction in VILI genesis.

## Introduction

Mechanical ventilation, a cornerstone of modern intensive care, is a lifesaving intervention for critically ill patients, and its appropriate use significantly improves survival. However, positive pressure ventilation (PPV) results in direct harm to the lungs, thereby impacting mortality especially in patients with acute respiratory distress syndrome (ARDS) (Hickling et al., 1990; Hickling et al., 1994; Amato et al.; Brower et al., 2000; Amato et al., 2015). Such harm has been termed ventilator-induced lung injury (VILI) and is classically thought to result from alveolar overdistension (volu-trauma) and/or atelectasis (atelect-trauma) along with the resultant biological injury (biotrauma) (Slutsky and Ranieri, 2013). Decades of research have highlighted the role of deleterious mechanical forces acting on the alveoli during positive pressure ventilation, resulting in surfactant inactivation, microvascular permeability, mechano-transduction, inflammation, and cellular failure (Dreyfuss and Saumon, 1998; Matthay et al., 2002; Vlahakis and Hubmayr, 2005). Additionally, PPV has been demonstrated to affect both the diaphragm (Goligher et al., 2018) and the brain (Bassi et al., 2021; Sparrow et al., 2021). A substantial body of literature has explored the role of airspace forces (viz. the airway pressures, Paw; tidal volume, Vt; and airflow) on the development of VILI, as well their respective impact on the pulmonary vasculature. More recently, experimental evidence has illustrated the contribution of the heart and its interaction with the lungs in the generation of lung injury through changes in both microvascular permeability and endothelial injury (Katira et al., 2017a; Katira et al., 2018; Katira et al., 2022).

Mechanical ventilation impacts the function of the right ventricle (RV) by inducing changes in the total intrathoracic (ITP), pleuro-pericardial and transpulmonary (PL) pressures (Magder and Guerard, 2012; Mahmood and Pinsky, 2018). Positive airway pressure (Paw) results in increased ITP, pleural (Ppl), pericardial and right atrial (Pra) pressures. These changes diminish the gradient for venous return, subsequently reducing the RV end-diastolic volume (RVEDV) and the RV wall stress (i.e., lowering preload). An increase in systemic venous pressure and stressed volume mitigate these effects (Nanas and Magder, 1992). When increase in ITP are associated with increase PL (i.e., Paw—Ppl), lung volume increases, affecting the pulmonary vascular resistance (PVR). Excessively high PL results in lung overinflation, markedly elevating the RV afterload because the alveolar pressure exceeds the pulmonary arterial and/or pulmonary venous pressures (West zones I and II) (Whittenberger et al., 1960). These collective changes ultimately determine the right ventricular (RV) output. The impact on the right heart during ARDS is typically considered secondary either to ventilator manipulation or increased pulmonary vascular resistance from hypoxemia, hypercarbia, and alveolar disease (Vieillard-Baron et al., 2016). While the heart is usually viewed as a victim in ARDS, it is not widely recognized whether the changes in right ventricular and pulmonary vascular dynamics-stemming from altered heart-lung interaction under mechanical ventilation, results in lung injury. This review aims to explain the role of heart-lung interactions and resultant changes in pulmonary vascular forces in the causation of VILI.

## Evidence from experimental VILI

### High stretch ventilation

The manifestation of pulmonary edema as a result of PPV was first demonstrated in the pivotal study by Webb and Tierney in 1974 (Webb and Tierney, 1974). This classic study utilized an *in vivo* rat model and tested ventilation with a range of combination of peak inspiratory (PIP) and positive end-expiratory pressures (PEEP)—PIP/PEEP (cmH_2_O) groups 14/0, 30/0, 30/10, 45/0 and 45/10. While the groups 14/0, 30/0 and 30/10 groups maintained stable compliance and gas exchange for an hour, most striking was the results in the 45/0 group, where all animals died within 30 min with pronounced pulmonary edema. Their lungs were notably heavy, demonstrated poor compliance and histology revealed marked perivascular and alveolar edema. Conversely, the application of 10 cmH_2_O PEEP to a high PIP (45 cmH_2_O) demonstrated a protective effect. The 45/10 group did not display any substantial change in lung weights, or alveolar edema, however they did display perivascular edema, which was also observed in 30/0 and 30/10 groups. The authors hypothesized that the alveolar edema in the 45/0 group resulted from increased surface forces; the expeditious alveolar edema occurred likely from rapid inactivation of surfactant, while replenishment lagged. Low positive end-expiratory pressure and high tidal volume have been shown to result in high surface forces leading to lower lung volume for the same transpulmonary pressure (Faridy et al., 1966). Perivascular edema was not consistently associated with alveolar edema and was theorized to result from decreased pressure in the perivascular space during inflation and resultant increased transmural pressure, a concept related to lung interdependence (Staub, 1963).

In subsequent decades, Dreyfuss and others further explored the same model, quantifying edema via extravascular lung water, and microvascular permeability utilizing dry lung weight and fractional albumin uptake (Dreyfuss et al., 1985). Within 5 min they demonstrated changes in microvascular permeability, and endothelial bleb formation, and notably it was not until 20 min that alveolar edema and diffuse alveolar damage (DAD) occurred. This demonstrated that endothelial injury preceded alveolar injury in this model. The application of PEEP was protective against increases in microvascular permeability and prevented edema formation. However infusion of dopamine to the animals with PEEP still resulted in alveolar edema, likely from increased vascular flow and pressure (Dreyfuss and Saumon, 1993). Despite these early findings highlighting the importance of cardiovascular interactions in the genesis and exacerbation of VILI, airspace forces have received the most attention, with research focused on the differentiation between barotrauma and volutrauma, as well as understanding the mechanism of alveolar shearing and/or overdistension in the causation of alveolo-capillary permeability.

**FIGURE 1:**
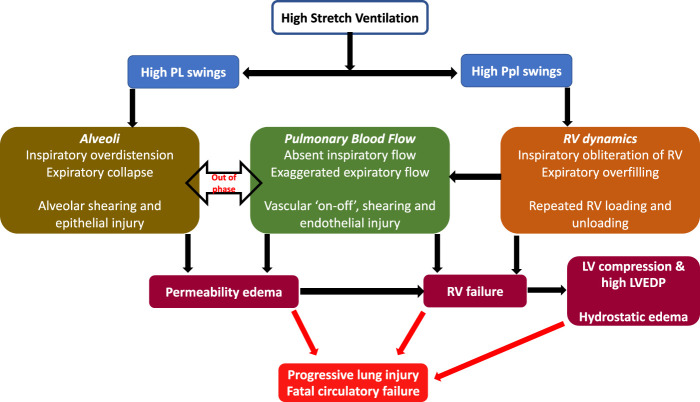
Cardiopulmonary interactions in VILI—High stretch ventilation from high Vt and low PEEP results in large swings of PL and Ppl. Swings in PL result in repeated opening and closing of alveoli and cyclical changes in PVR, while swings in Ppl cause cyclical changes in RV preload. These together result in large swings of pulmonary blood flow, a vascular ‘on-off’ phenomena and endothelial shearing. This results in increased microvascular permeability and progressive lung injury. RV failure results from vascular injury, repeated loading and unloading of RV as well as lung injury. RV failure leads to LV compression and high LVEDP and consequent contribution to increased lung water (hydrostatic edema). Abbreviations: Vt Tidal Volume; PEEP Positive End-Expiratory Pressure; PL Transpulmonary pressure; Ppl Pleural pressure; RV Right Ventricle; PVR Pulmonary Vascular Resistance; LVEDP Left ventricular end-diastolic failure.

Given the precipitous onset of edema and rapid death (due to cardiovascular collapse), it was recently hypothesized that the injury in this seminal model was likely from adverse heart lung interactions under mechanical ventilation (Katira et al., 2017a). In addition to measuring lung permeability, authors investigated the heart using echocardiography, and measurements of ventricular pressures. The hemodynamic effects in the 45/0 group were most pronounced. During inspiration in the 45/0 the right ventricle was markedly underfilled, in contrast the 45/10 group demonstrated constant RV filling during both inspiratory and expiratory cycles. This absence of right ventricle filling led to total abolition of RV stroke volume and cessation of pulmonary blood flow during inspiration. This was followed by exaggerated RV output and flow during expiration, resulting in cyclic ‘on-off’ pulmonary blood flow with each respiratory cycle (Figure 1) (Katira et al., 2017b). Furthermore, the PVR during inspiration (although not measurable due to absent flow) must be substantial, given the high inspiratory PL and likely alveolar capillary compression, further exacerbating the swings in RV output. As a result, lungs were in zone I condition during inspiration and received accelerated and potentially shearing blood flow during expiration when the lungs underwent collapse (negative expiratory PL). Vascular shearing can lead to endothelial injury from the well documented phenomena of capillary stress failure, which in turn leads to increased microvascular permeability. Moreover, the repeated loading and unloading of the right ventricle, combined with escalating vascular and alveolar injury, led to RV dilation (increased RV/LV ratio) and subsequent failure. RV dilation also led to left ventricular (LV) encroachment and increased LV filling pressure (Pinsky, 2016) (ventricular-ventricular interaction), in turn contributing to increased lung water.

Conversely the 45/10 group had decreases in both RV and LV volume and output, but this remained constant throughout the respiratory cycle and through the experiment, thus explaining the protective effect of lower Vt and PEEP. The application of 10 cmH_2_O PEEP increased the Ppl and likely Pra, thereby reducing cardiac output while maintaining a positive end-expiratory PL (preventing lung collapse). Lower Vt resulted in lower inspiratory PL and hence a reduced RV afterload, together these changes provided a stable variation in pulmonary blood flow likely avoiding vascular shearing, permeability, and RV failure.

### Abrupt deflation

**FIGURE 2:**
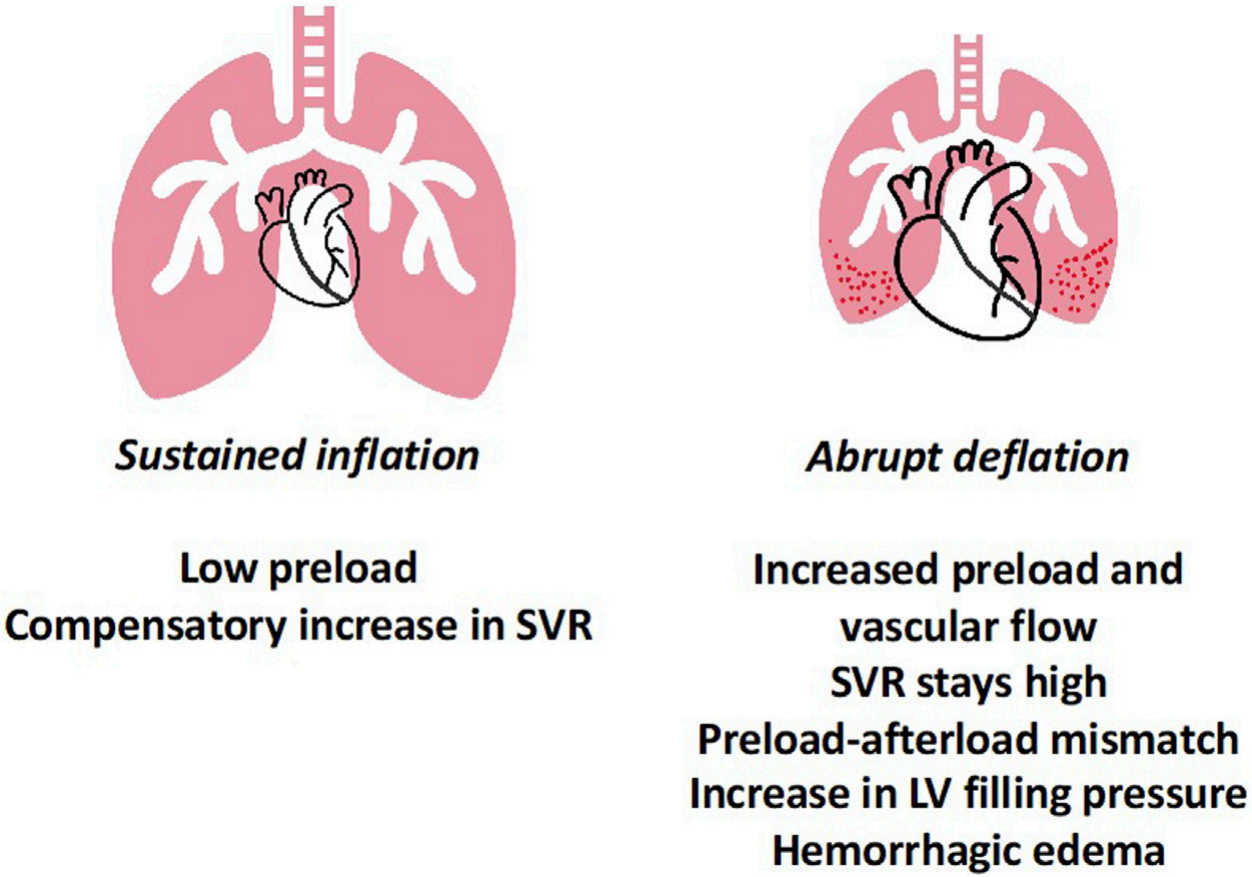
Lung Deflation Injury—sustained inflation at high PEEP results in lower preload and compensatory increase in systemic vascular resistance to maintain blood pressure. Upon abrupt deflation, preload increases, however the SVR remains high, leading in preload-afterload mismatch for the left ventricle. Together this results in high LVEDP and along with abruptly increased pulmonary blood flow possibly leads to capillary stress failure and permeability edema. Abbreviations: SVR Systemic Vascular Resistance; PEEP Positive End-Expiratory Pressure; LVEDP Left Ventricular End-Diastolic Pressure.

In another experimental set-up, using healthy rats, it was demonstrated that abruptly deflating the lung after sustained inflation from clinically relevant pressure and volume (PEEP 12 cmH_2_O and Vt 6–7 mL/kg), resulted in hypoxemia, poor lung compliance, protein and water leak into the alveoli, as well as histological evidence of injury (Figure 2) (Katira et al., 2018). The microvascular damage as measured by Evans blue dye, was shown to be minimal prior to deflation and increased progressively after deflation, peaking within 5–10 min. Electron microscopy revealed endothelial injury, which provided the biological basis of this leak; the leak was prevented by gradual deflation. Importantly, the injury began with endothelial damage, suggesting a cardiovascular mechanism. Upon investigation into the cardiovascular dynamics using echocardiography and intraventricular pressure measurements, it was observed that with increase in PEEP (during the inflation limb), preload and cardiac output decreased. Upon deflation, preload, and RV output abruptly increased, faced with abruptly increased PVR, which was associated with a sudden increase in left ventricular end-diastolic pressure (LVEDP). The physiological events can be divided into three parts sustained inflation, abrupt deflation and post deflation. During inflation, as the preload and cardiac output decrease, arterial pressure reduces and results in compensatory arterial vasoconstriction and increased LV afterload. The abrupt deflation leads to an acute increase in preload and output, which had two-fold effect—first, an acute increase in pulmonary blood flow and second, acute LV decompensation because the abrupt increase in LV preload was met with high LV afterload (preload-afterload mismatch). These two events together likely gave rise to high pulmonary capillary pressure, causing endothelial injury and microvascular leak. In the post deflation phase, vascular injury and pulmonary edema contribute to ongoing lung injury, increasing PVR and leading to RV failure. Additionally, pretreating animals with sodium nitroprusside abolished the LV preload-afterload mismatch likely through systemic vasodilation, and reduced lung injury for similar pattern of inflation followed by deflation.

A similar experimental plan was performed in a porcine model (Katira et al., 2022), wherein the increase in LVEDP was not observed. However, a single abrupt deflation from high PEEP resulted in increased lung water, accompanied by increased pulmonary blood flow, elevated PVR and hypoxemia. In contrast, gradual deflation resulted in gradual increase in blood flow, lower PVR and improved oxygenation. Additionally, repeated short lung deflations from clinically relevant levels of PEEP resulted in pulmonary edema, and a trend towards protein leakage and inflammation. These changes were accompanied by worsening respiratory system compliance, increased RV afterload and hypoxemia. Taken together, these results from *in-vivo* experiments suggest that high pulmonary vascular flow and high microvascular pressure (either from forward or backward pressure) interact with airspace forces in the genesis of VILI(28).

### *In vitro* studies

Several key studies provide insight into what components of pulmonary vascular dynamics impact lung injury, using the isolated rabbit heart-lung preparation (Broccard et al., 1998; Broccard et al., 1999; Hotchkiss et al., 2000; Hotchkiss et al., 2001). Lungs were submitted to varying levels of perfusion while the same injurious pattern of ventilation was used (Broccard et al., 1998). It was observed that perfusion amplitude contributed to a reduction in lung compliance, as well as the formation of hemorrhage and edema. While it initially appeared that the perfusion pressure gradient was the principal determinant, whether the culprit in these interactions was pulmonary blood flow or pressure was further explored by varying the airway pressures to allow variation in pulmonary arterial pressures while holding blood flow constant (Broccard et al., 1999). Their results indicated higher mean Paw had a greater impact than tidal excursion in the development of lung permeability and hemorrhage, especially because higher mean Paw was associated with higher pulmonary artery pressure, demonstrating modifications of vascular pressure could impact the severity of ventilator induced lung injury. The authors suggested that higher upstream pressure in the pulmonary vasculature, i.e., Pulmonary artery to alveolar pressure gradient, results in hemorrhagic pulmonary edema (Marini et al., 2003). Furthermore, the role of number of respiratory cycles was also explored—with lungs ventilated at the same peak pulmonary artery pressures formed less edema and perivascular hemorrhage when ventilated at lower frequency (Hotchkiss et al., 2000). These findings indicated that not only the characteristics of the breath are of importance but the ventilatory frequency and the cyclical variation of pulmonary artery pressure too are contribute to repeated vascular strain and stress.

In another setting, addressing the intersection of pulmonary blood flow and pulmonary capillary pressure (Pcap), it was noted that lungs subjected to high pulmonary blood flow, with or without high Pcap developed progressive weight gain, edema formation, hemorrhage, and increased filtration (Lopez-Aguilar et al., 2006). Interestingly, lungs subjected to high Pcap with low blood flow exhibited much less lung injury similarly to the group of low Pcap and low blood flow. The authors concluded that high blood flow when coupled with cyclical inflation of lungs is likely to increase the shear stress and wall tension in pulmonary capillaries - specifically extra-alveolar capillaries during inspiration and alveolar capillaries during expiration. Such increases in capillary stressors will result in endothelial failure, leak, and inflammation. Moreover, in a model of chronic pulmonary arterial hypertension (PAH), injurious ventilation strategy resulted in lower lung injury, inflammation, and hypoxia, while normal animals subjected to similar ventilation demonstrated hypoxia, poor compliance, increased lung weight and higher cytokine expression (Kornecki et al., 2008). This could be due to increased basement membrane thickness in the presence of PAH, rendering the alveolar capillary membrane more resistant to mechanical injury from either airspace or vascular forces. In contrast, adding negative pressure to high stretch ventilation resulted in greater lung injury compared to positive pressure alone, partly due to increased pulmonary perfusion in the negative pressure group (Dreyfuss et al., 1988).

Aforementioned experiments highlight the significant role of pulmonary artery pressure, pulmonary blood flow and LV filling pressure on lung microvascular health. This perspective is further reinforced by observations showing a disproportion of vascular injury occurring in the dependent lung, which receives most of the lung’s blood flow (Broccard et al., 2000). These areas thus may be more vulnerable to shearing stresses within the vascular endothelium. Pulmonary vascular dynamics are directly impacted by the pattern of mechanical ventilation; injurious strategies lead to adverse cardiopulmonary (CP) interactions, microvascular injury, and alveolar edema, while protective patterns (e.g., low Vt and PEEP) may result in favorable CP interactions and consequent less or no VILI. It is therefore to be noted that vascular forces work in conjunction with airspace forces, and both together impact the genesis of VILI (Figure 1) (Hotchkiss et al., 2001). It is well understood that high airway pressure may result in increased alveolar epithelial permeability and even gas leak into the circulation (Egan et al., 1976; Egan, 1980; Egan, 1982). Cyclic opening and closing with high Vt results in surfactant dysfunction, increased alveolar stress in the regions of collapse lung surrounded by overdistended regions, and alveolar shearing (Tremblay and Slutsky, 2006).

## Translational implication

The role of CP interactions and vascular forces in VILI suggests cardiovascular targets for lung protection exist, in addition to pulmonary ones (e.g., plateau pressure, driving pressure, Vt, etc.). Monitoring pulmonary vascular dynamics through techniques such as echocardiography or pulmonary artery catheter measurements could help identify patients at risk of lung injury. Parameters such as high pulmonary artery pressure, cyclic alteration in pulmonary blood flow, and high LV filling pressure can all be measured or assessed at various airway pressures, informing the CP interaction at the bedside (Vieillard-Baron et al., 2016). It was noted that increase in tidal volume led to high transpulmonary pressure swings which in turn resulted in cyclic alteration in pulmonary blood flow (Vieillard-Baron et al., 1999). Furthermore, in post operative cardiac patients, under passive ventilation an increase in Vt (and driving pressure) resulted in higher non-zone III conditions (Slobod et al., 2022). Therefore, by testing different levels of tolerable PEEP and Vt (analogous to PEEP titration for optimal lung mechanics) it is possible to understand the impact on right heart and pulmonary hemodynamics and possibly distinguish the effects of mechanical ventilation from existing lung injury. Modulation of mechanical ventilation to optimize the CP interactions may help reduce forces on both sides of alveolar capillary membrane. Moreover, limiting lung deflations due to ventilator disconnections not only improves lung function but may also reduce lung injury from atelectasis and vascular forces (Maggiore et al., 2003; Maggiore et al., 2013; Katira et al., 2022).

Furthermore, patients at risk of endothelial injury (e.g., sepsis, etc.), could benefit from strategies aimed at lowering pulmonary artery pressure and flow, as well as paying careful attention to use of vasoactive medications as they can augment pulmonary macro and microvascular stressors. It has been shown that restricting fluid infusion in patients with ARDS improves outcomes (Wiedemann et al., 2006; Semler et al., 2016) and increased lung water is associated with severity and poor outcomes in ARDS (Craig et al., 2010; Kushimoto et al., 2013). Concurrently, the presence of RV failure in ARDS, although multifactorial, is associated with poor outcomes (Zochios et al., 2017). Therefore, balancing the use of fluids, vasoactive medications and ventilatory strategies to support the RV enough without adversely impacting pulmonary vascular dynamics (and microvascular stressors) may provide key strategies to either prevent or limit ongoing lung injury.

## Future directions

Translational research is needed to study the tangible impact of adverse cardiopulmonary interactions, as seen during abrupt deflation, ventilator weaning, fluid overload, high stress (driving pressure) ventilation in the preclinical models of ARDS, heart failure and sepsis. Together this might provide additional insight into the failure of clinical trials deploying high PEEP or recruitment. Additionally, incorporating ventilatory strategies like those used at the bedside unlike the extreme ones used to elicit mechanisms in the classic models, could further improve the translational capability. Moreover, studies exploring heart and lung protective ventilation in critically ill patients, impact of pronation of pulmonary vascular macro and micro dynamics, and optimization of CP interactions under ECMO, are additional research avenues in this line of investigation.

## Summary

Both experimental and clinical evidence highlight the occurrence of adverse cardiopulmonary interaction during injurious ventilation and provide evidence of vascular mediated lung injury. As our understanding of endothelial damage in VILI grows, the role of macro and microvascular dynamics, particularly pulmonary blood flow and pressure seems pivotal in VILI genesis. These observations also provide insight into the ventilator related mechanisms of RV failure and suggest the need to develop heart and lung protective ventilatory strategies, starting with careful observations of bedside hemodynamics coupled with respiratory mechanics and gas-exchange.
